# P-644. Antibiotic Prescription Rate for Viral Respiratory Infections After a Viral Care Package Intervention and Provider Education in the Urgent Care Setting

**DOI:** 10.1093/ofid/ofaf695.857

**Published:** 2026-01-11

**Authors:** Kayla M Backus, Nicholas Torney, Cynthia Nichols

**Affiliations:** Medical University of South Carolina, Holland, MI; Munson Medical Center, Traverse City, MI; Munson Medical Center, Traverse City, MI

## Abstract

**Background:**

Antibiotics are often prescribed for patients diagnosed with Tier 3 viral respiratory infections (e.g., acute bronchitis, upper respiratory tract infections), despite strong recommendations against their use. The aim of this study was to assess if the implementation of a viral care package plus a one-time provider education (VCP+E) was associated with a reduction in antibiotic prescriptions for Tier 3 infections in the urgent care (UC) setting.

**Methods:**

Viral care packages (VCPs) with over-the-counter products were made for one Munson Healthcare UC and developed into four age-appropriate packages for patients 2 years and older. They were dispensed from 11/1/2024-1/31/2025 following a provider education session on the appropriate use of antibiotics for Tier 3 infections. Patients diagnosed with a Tier 3 infection only were eligible to receive a VCP. Outcomes were assessed pre (10/1/2023-4/30/2024) and post (2/1/2025-4/30/2025) VCP+E implementation. The primary outcome assessed antibiotic prescription rate for patients with Tier 3 infections, compared to a combined group of 3 UCs without VCP+E. Secondary outcomes assessed antibiotic prescription rate for all encounters, rate of antibiotic prescriptions within 7 days of receiving a VCP, and patient and provider satisfaction through a survey.

**Results:**

379 VCPs were dispensed during the study timeframe. The rate of antibiotic prescriptions for Tier 3 infections was lower after VCP+E implementation (13.9 vs. 9.1%, p< 0.001), with no change in the group without VCP+E during the same time period (35.2 vs. 37.2%, p=0.529). This change was largely driven by a reduction in prescriptions for acute bronchitis in the VCP+E group (44.1 vs. 25%, p< 0.001). Overall rate of antibiotic prescriptions for any indication was higher in both groups after implementation (VCP+E= 30.4 vs. 34.1%, p < 0.001; no E+VCP= 41.8 vs. 45.9%, p< 0.001). Within 7 days of receiving a viral care package, 7.9% of patients received an antibiotic. Approximately 10% (n=36) of patients who received a VCP responded to a survey with average patient satisfaction being 9.6/10.

**Conclusion:**

A provider-driven VCP intervention plus a one-time provider education was associated with a decrease in antibiotic prescriptions for Tier 3 respiratory infections in the UC setting.

**Disclosures:**

All Authors: No reported disclosures

